# Contigs directed gene annotation (ConDiGA) for accurate protein sequence database construction in metaproteomics

**DOI:** 10.1186/s40168-024-01775-3

**Published:** 2024-03-19

**Authors:** Enhui Wu, Vijini Mallawaarachchi, Jinzhi Zhao, Yi Yang, Hebin Liu, Xiaoqing Wang, Chengpin Shen, Yu Lin, Liang Qiao

**Affiliations:** 1 Department of Chemistry, and Shanghai Stomatological Hospital, Fudan University, Shanghai, 200000 China; 2 School of Computing, College of Engineering, Computing and Cybernetics, The Australian National University, Canberra, ACT 2600 Australia; 3 Flinders Accelerator for Microbiome Exploration, College of Science and Engineering, Flinders University, Bedford Park, SA 5042 Australia; 4 Shanghai Omicsolution Co., Ltd, Shanghai, 200000 China

**Keywords:** Taxonomic annotation, Metaproteomics, Metagenomics, Microbiota, Mass spectrometry

## Abstract

**Background:**

Microbiota are closely associated with human health and disease. Metaproteomics can provide a direct means to identify microbial proteins in microbiota for compositional and functional characterization. However, in-depth and accurate metaproteomics is still limited due to the extreme complexity and high diversity of microbiota samples. It is generally recommended to use metagenomic data from the same samples to construct the protein sequence database for metaproteomic data analysis. Although different metagenomics-based database construction strategies have been developed, an optimization of gene taxonomic annotation has not been reported, which, however, is extremely important for accurate metaproteomic analysis.

**Results:**

Herein, we proposed an accurate taxonomic annotation pipeline for genes from metagenomic data, namely contigs directed gene annotation (ConDiGA), and used the method to build a protein sequence database for metaproteomic analysis. We compared our pipeline (ConDiGA or MD3) with two other popular annotation pipelines (MD1 and MD2). In MD1, genes were directly annotated against the whole bacterial genome database; in MD2, contigs were annotated against the whole bacterial genome database and the taxonomic information of contigs was assigned to the genes; in MD3, the most confident species from the contigs annotation results were taken as reference to annotate genes. Annotation tools, including BLAST, Kaiju, and Kraken2, were compared. Based on a synthetic microbial community of 12 species, it was found that Kaiju with the MD3 pipeline outperformed the others in the construction of protein sequence database from metagenomic data. Similar performance was also observed with a fecal sample, as well as in silico mixed datasets of the simulated microbial community and the fecal sample.

**Conclusions:**

Overall, we developed an optimized pipeline for gene taxonomic annotation to construct protein sequence databases. Our study can tackle the current taxonomic annotation reliability problem in metagenomics-derived protein sequence database and can promote the in-depth metaproteomic analysis of microbiome. The unique metagenomic and metaproteomic datasets of the 12 bacterial species are publicly available as a standard benchmarking sample for evaluating various analysis pipelines. The code of ConDiGA is open access at GitHub for the analysis of microbiota samples.

Video Abstract

**Supplementary Information:**

The online version contains supplementary material available at 10.1186/s40168-024-01775-3.

## Background

The human body is composed of both human cells and many different microorganisms. Through host-microbiota interactions, microbes are closely associated with various diseases, including luminal diseases, immune diseases, metabolic diseases, and neurodegenerative diseases. To understand the role of microorganisms in human health and disease, it is necessary to characterize the changes in composition as well as the functional dynamics of microbiota. With the development of next-generation sequencing (NGS) techniques, metagenomics has greatly facilitated the study of microbiota [1, 2]. While metagenomics provides information on the composition and functional potential of microbiome, the method cannot reveal proteins actually expressed in the microbiome [3, 4]. Proteins, as biomolecules performing various functions within organisms, should be identified and quantified directly for microbiota function study.

During the past years, mass spectrometry (MS)-based metaproteomics has been emerging as a powerful approach to understanding the functions of microbial communities [5–9]. Compared to traditional proteomics of a single organism or simple mixtures, metaproteomics of microbiota faces challenges of high complexity and taxonomic diversity, wherein dozens or even hundreds of species can present in a sample with wide dynamic range variations, making the proteomic analysis extremely difficult [3, 10]. In shotgun proteomics, peptide identification relies on matching tandem mass spectra with protein sequences in a database. A crucial step in metaproteomics is to choose a suitable protein sequence database. An incomplete database can result in the missing of some key proteins, while a database with too large search space can result in limited detection sensitivity and high false discovery rates [11–13].

To date, the database-building strategies in metaproteomics include mainly (i) refining public proteome sequence databases, e.g., NCBInr [14] and UniProtKB/Swiss-Prot [15], using mass spectrometric data [16–20]; (ii) filtering public proteome sequence databases using taxonomic information by 16S rRNA sequencing [13, 21, 22]; and (iii) constructing sample-specific proteome sequence databases by whole-genome sequencing [10, 12, 13]. Among the different strategies, sample-specific database construction by metagenomics sequencing has been considered the best choice and employed in many metaproteomic studies [12, 23–26], wherein the databases contain only protein sequences specific to samples and hence can offer the best-fit search space to explore the protein expression of a particular microbiota and to improve the overall proteome coverage. However, accurate taxonomic annotation of metagenomics data, which acts as a key role in the construction of protein sequence databases, remains a challenging task [10, 26]. Currently, there are three major taxonomic annotation strategies, i.e., read-level annotation, gene-level annotation, and contig-level annotation. Different approaches for taxonomic annotation of genes result in significantly divergent results in the downstream metaproteomic studies [12, 24]. There is, to date, no consensus on a robust and reliable approach for gene taxonomic annotation.

## Results

### Optimization of taxonomic annotations of genes based on simulated microbial communities

**Fig. 1:**
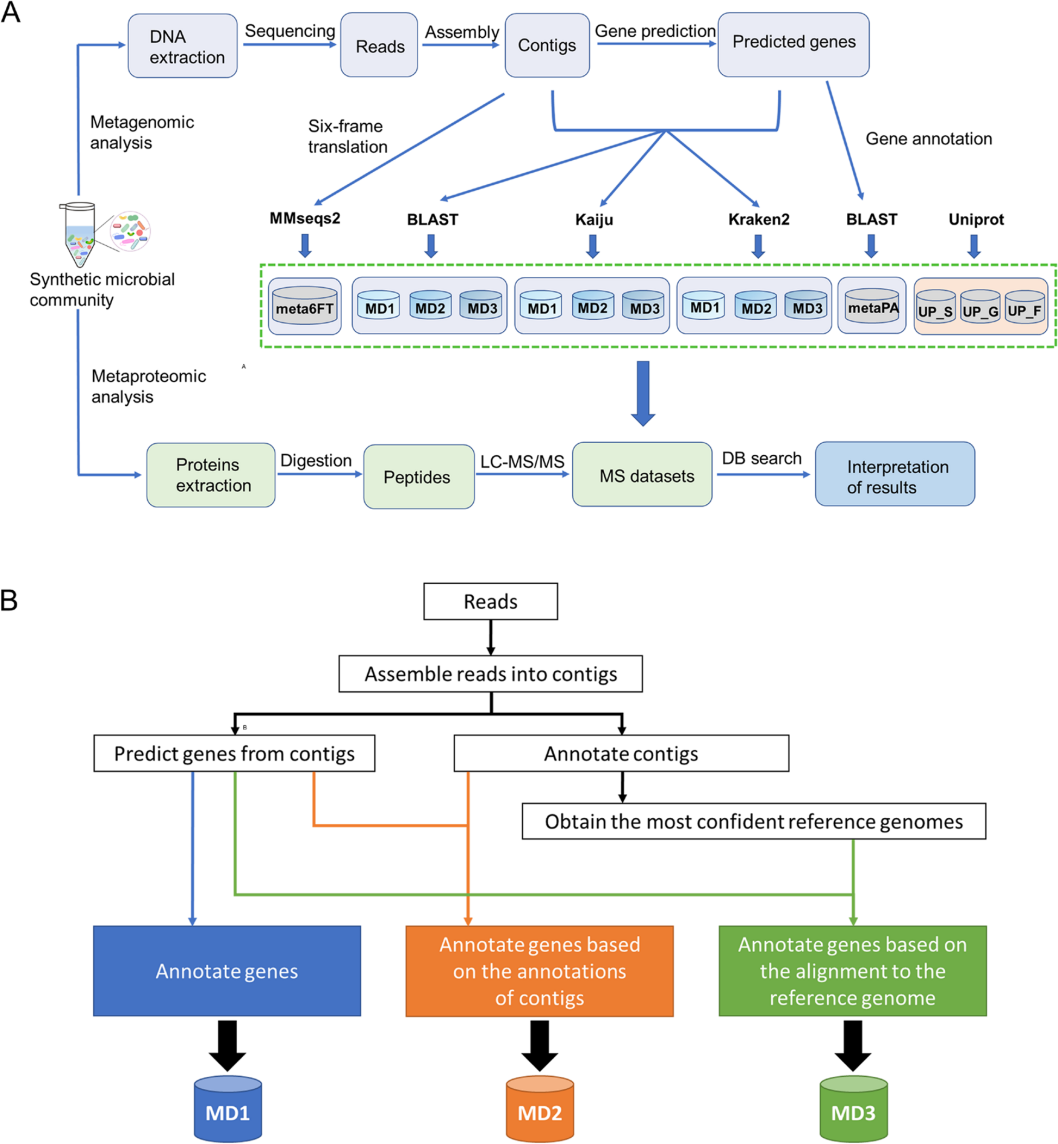
**A** Benchmarking different protein sequence databases from metagenomics or from Uniprot reference proteome using a synthetic microbial community of 12 species. **B** Pipeline of three annotation strategies (MD1, MD2, and MD3)

In this work, we developed an accurate gene taxonomic annotation strategy, namely contigs directed gene annotation (ConDiGA, MD3, Fig. 1). Contigs assembled from metagenomic sequencing results were firstly annotated. Afterwards, the most confident species were selected, and a gene-level annotation was performed based on the reference genome of the selected species. Two commonly used annotation strategies were compared with the ConDiGA, including the MD1 pipeline of annotating genes directly against the whole bacterial genome database and the MD2 pipeline of taking the contig taxonomic annotation information as the corresponding gene taxonomic information (Fig. 1). In all three pipelines, different annotation tools, including BLAST [27], Kaiju [28], and Kraken2 [29], were compared. Referred to the work by Tanca et al. [24], we also built a meta6FT database by six-frame translation of the contigs using MMseqs2 [30], as well as a metaPA database where the amino acid sequences of the predicted genes were annotated using the UniProtKB/TrEMBL database using Protein BLAST.

We mixed 12 known bacterial species to derive a synthetic microbial community (Supplementary Table 1) to benchmark the different protein sequence database construction strategies. We also included the Uniprot reference proteome at the species level (UP-S), genus level (UP-G) and family level (UP-F) for comparison. The detailed information in terms of the number of genes annotated and species annotated of the protein sequence databases built by the three pipelines are shown in Supplementary Data 1, Supplementary Figs. 1, 2 and 3, and Supplementary Table 2. The Uniprot reference proteomes of the 12 species are shown in Supplementary Table 3. From the perspective of database annotation, MD1_BLAST acquired the highest annotation rate of over 90%. However, MD1_BLAST also had a high error rate in annotation (Supplementary Table 2), and 122 species were identified by the MD1_BLAST. The MD1 pipeline with all three annotation tools led to a significant number of false annotations at the species level. MD1_Kaiju identified 157 species, and MD1_Kraken2 identified 52 species. MD2 was more accurate than MD1 in taxonomic annotation at the species level. MD2_BLAST identified 77 species, MD2_Kaiju identified 121 species, and MD2_Kraken2 identified 33 species. The MD3 is most accurate in taxonomic annotation at the species level. Since the genes were annotated based on a refined set of species, MD3 not only had a higher recall on gene annotations but also was robust against mis-assemblies. All the annotation tools with MD3 led to only 12 species being identified. However, it should be noted that MD3 with BLAST had one mis-annotation, wherein *Klebsiella variicola* was annotated instead of *Lactobacillus acidophilus*. Two close relatives of *Klebsiella variicola*, i.e., *Klebsiella aerogenes* and *Klebsiella pneumoniae*, were included in the synthetic community, which can explain the mis-annotation by MD3 with BLAST.

**Fig. 2:**
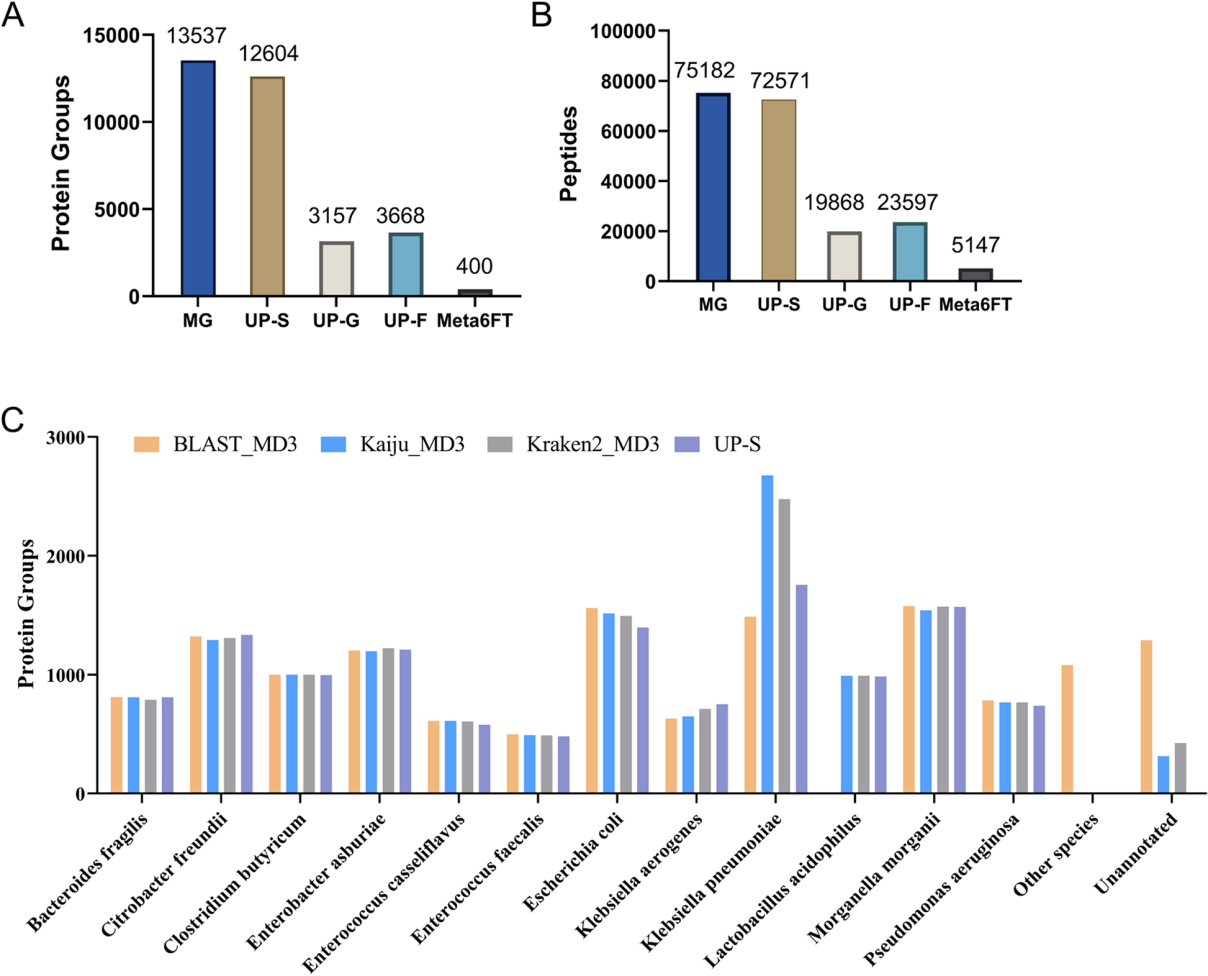
Numbers of **A** protein groups and **B** peptides identified for the 12 target species from the synthetic microbial community using different databases. **C** Numbers of protein groups identified from each species using the databases constructed with the MD3 pipeline and the UP-S reference database

We then compared the different protein sequence databases by the different annotation strategies on the analysis of the metaproteomic data of the synthetic microbial community. It should be noted that the protein sequences in all the databases by MD1, MD2, and MD3 were the same, and only the annotation results were different, because the different pipelines shared the same gene prediction strategy. The proteins without annotation information at species level were labeled as unknown in the databases. Protein group identification results are shown in Table 1 and Supplementary Data 2. The identification results using the UP-S were considered as the reference. Generally, the numbers of protein groups identified for the 12 target species using the MD3-based databases were significantly higher than those using the MD1- and MD2-based databases. As for the annotation tools, BLAST performed better overall, but Kaiju-MD3 managed to become the most efficient strategy, which led to the identification of 13,537 protein groups for the 12 target species. In contrast, the UP-S only led to 12,604 protein groups identified for the 12 target species. Figure 2A and B show the numbers of protein groups and peptides identified for the 12 target species using the databases of Kaiju with the MD3 pipeline (shown as MG), UP-S, UP-G, UP-F, and Meta6FT. The results by the UP-G, UP-F, and Meta6FT databases were not comparable to those by the MD3-based databases. For further analysis, we compared the protein groups identified for each species using MD3_Kaiju, MD3_BLAST, MD3_Kraken2, and UP-S (Fig. 2C). The numbers of protein groups assigned to each species using the MD3-based databases were basically consistent with those using the UP-S except for *K. pneumonia* and *Lactobacillus acidophilus*. The number of protein groups identified for *K. pneumonia* using MD3_Kaiju and MD3_Kraken2 was higher than that using UP-S. UP-S database was constructed using the reference stains, which can be different from the strains in our synthetic microbial community. By metagenomic sequencing, it is possible to identify some proteoforms with sequence variations not included in UP-S. Based on the benchmarking with the synthetic microbial community of 12 species, it was demonstrated that the MD3 pipeline is most suitable for annotating genes in the construction of metagenomics-derived database for metaproteomic analysis, and the MD3 strategy plus Kaiju served the best among all the tested methods.

**Table 1:** Identification results of protein groups for the 12 target species from the synthetic microbial community using different databases

	BLAST			Kaiju			Kraken2			MetaPA	Meta6FT	UP-S	UP-G	UP-F
Species name	MD1	MD2	MD3	MD1	MD2	MD3	MD1	MD2	MD3	——	——	——	——	——
*Bacteroides fragilis*	808	805	**810**	247	786	**809**	800	809	**788**	22	4	808	39	410
*Citrobacter freundii*	1266	1294	**1322**	214	557	**1290**	103	113	**1309**	293	30	1335	361	8
*Clostridium butyricum*	1078	1057	**1000**	327	785	**1000**	1082	1082	**1000**	226	18	996	1	286
*Enterobacter asburiae*	1198	1224	**1204**	85	294	**1197**	602	1141	**1221**	233	29	1210	222	1
*Enterococcus casseliflavus*	80	134	**612**	133	597	**612**	136	320	**607**	81	4	578	158	200
*Enterococcus faecalis*	490	489	**498**	137	319	**491**	476	491	**489**	134	5	480	286	362
*Escherichia coli*	1476	1266	**1559**	208	377	**1515**	284	486	**1495**	845	76	1397	571	685
*Klebsiella aerogenes*	624	564	**630**	496	696	**649**	606	791	**713**	292	91	751	8	10
*Klebsiella pneumoniae*	1505	891	**1488**	377	875	**2676**	183	209	**2477**	904	591	1756	192	254
*Lactobacillus acidophilus*	987	954	**0**	948	991	**991**	984	991	**991**	346	20	985	314	359
*Morganella morganii*	1592	1595	**1577**	307	1259	**1540**	1594	1597	**1572**	470	11	1569	932	999
*Pseudomonas aeruginosa*	772	770	**783**	55	102	**767**	57	182	**767**	54	9	739	73	94
*Target 12 species*	*11,876*	*11,043*	***11,483***	*3534*	*7638*	***13,537***	*6907*	*8212*	***13,429***	*1734*	*400*	*12,604*	*3157*	*3668*
Other species	869	644	**1081**	241	259	**0**	144	45	**0**	8218	2496	——	2842	2336
Unannotated	1107	2165	**1288**	10,077	5955	**315**	6801	5595	**423**	3900	888	——	——	——
Total	13,852	13,852	13,852	13,852	13,852	13,852	13,852	13,852	13,852	13,852	3784	12,604	5999	6004

### Benchmarking the performance of ConDiGA on fecal sample

**Fig. 3:**
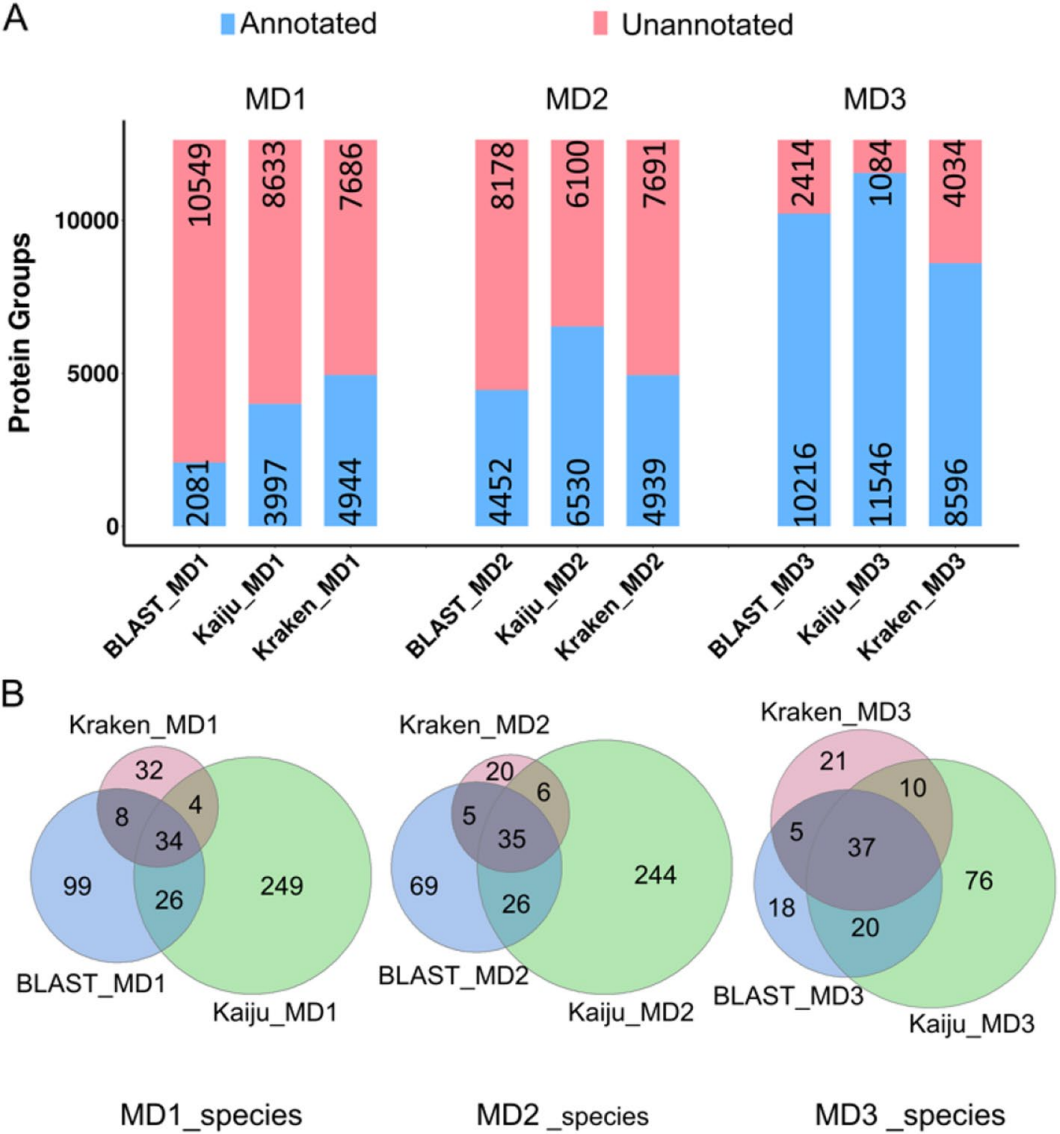
**A** Bar plots of the numbers of protein groups with and without taxonomic annotation identified from the stool sample using the MD1, MD2, and MD3 pipeline-based databases. **B** Venn diagrams of the species identified from the stool sample using the MD1, MD2, and MD3 pipelines-based databases

To test the performance of the ConDiGA pipeline in real microbial communities, a stool sample from a healthy volunteer was collected and processed for metagenomic and metaproteomic analyses. Protein sequence databases were constructed from the metagenomic sequencing results using the MD1, MD2, and MD3 pipelines with the annotation tools of BLAST, Kaiju, and Kraken2. The detailed information in terms of the number of genes annotated and species identified of the protein sequence databases are shown in Supplementary Data 3 and Supplementary Table 4. From the perspective of taxonomic annotation, MD3_Kaiju showed the highest annotation rate of over 60%, followed by MD2_Kaiju and MD3_BLAST with 51.41% and 44.67%, respectively. As depicted in Fig. 3A and Supplementary Data 4, the numbers of identified protein groups with taxonomic annotation employing the MD3 pipeline with all three annotation tools tremendously exceeded the ones with the MD1 and MD2 pipeline. The best performance was again obtained with the MD3 pipeline and the Kaiju-based annotation, where > 90% of the identified protein groups were successfully annotated at the species level. When considering the species identified by the different pipelines, the MD3 showed the best consistency with the different annotation tools, i.e., BLAST, Kaiju, and Kraken2. With the MD3 pipeline, MD3_Kaiju identified the largest number of species (143 species) and covered the majority of the species identified by MD3_BLAST (71.3%, 57/80) and MD3_Kraken2 (64.4%, 47/73) (Fig. 3B).

**Fig. 4:**
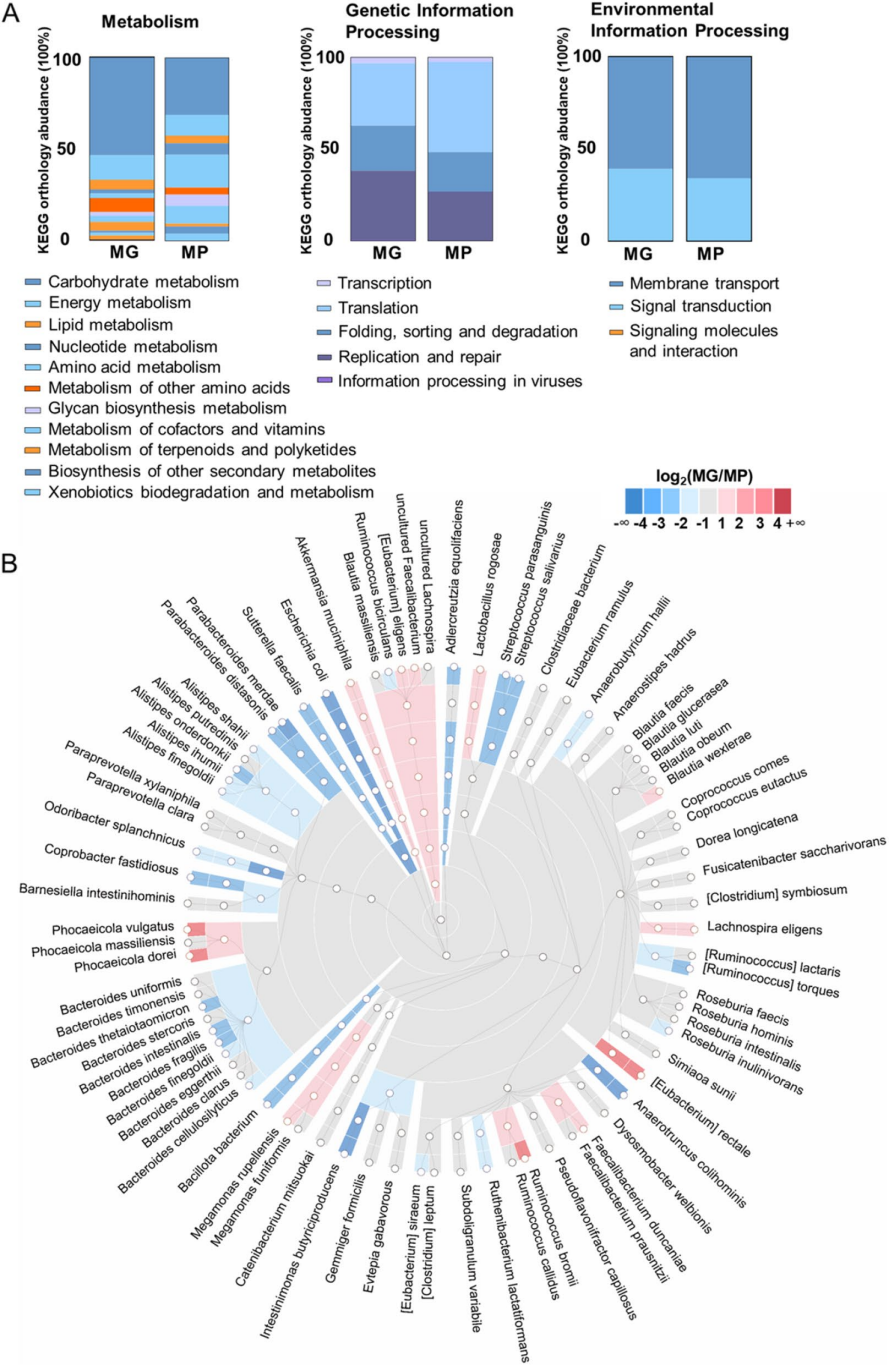
Comparative analysis of function and taxonomy at the metagenomic (MG) and metaproteomic (MP) levels. **A** KEGG functional annotation by MG and MP. The labels for the function terms are displayed in an order the same as the stacked bar charts. **B** Taxa abundances by MG and MP. Colors indicate the log_2_ ratio of relative abundance measured by MG/MP

To further analyze the stool sample, we conducted a comparative investigation of functional and taxonomic features at metagenomic (MG) and metaproteomic (MP) levels. Metagenomic and metaproteomic results based on the MD3_Kaiju were annotated using KEGG Orthology (KO) through the GhostKOALA website. Figure 4A shows the comparison of the KO functional annotation between the MG and MP in terms of metabolism, genetic information processing, and environmental information processing. Notably, the KO annotation results by the two meta-omics methodologies were mostly consistent. Carbohydrate metabolism, (protein forming) amino acid metabolism, and energy metabolism were the top three abundant metabolism categories according to MP, while MG suggested carbohydrate metabolism, metabolism of other amino acids, and energy metabolism as the top three. This result highlighted the divergence between metagenomics and metaproteomics in revealing functional potential of microbiota.

The relative taxonomic abundance at the metaproteomic level was computed by summing the quantitative information of all the identified peptides of each species, and the relative taxonomic abundance at the metagenomic level was calculated by counting the number of genes assigned to each species (Supplementary Data 5). The cladogram in Fig. 4B depicts the relative abundance discrepancies between MP and MG by calculating the log_2_ MG/MP abundance ratio. The results showed that the abundance differences existed at different taxa levels. MP and MG abundance were generally consistent at order level and levels beyond order, while their distinguishment appeared frequently below order level. As an example, in the Bacteroidaceae family, the family-level MG/MP ratio was close to one, while the MG abundance was higher in *Phocaeicola* genera and the MP abundance was higher in *Bacteroides* genera*.* These results indicated that within the same family, the relative abundance of different species by metagenomics and metaproteomics can display heterogeneity. The relative abundance by MG is closely related to the cell copy of a species, while the relative abundance by MP shows the total protein amount of a species. The relative abundance discrepancies between MP and MG have also been reported by Tanca et al. [31]*.*

### *Benchmarking on *in silico* mixed metagenomic and metaproteomic data*

To further demonstrate the annotation sensitivity and accuracy of the ConDiGA strategy, we computationally mixed the data of the synthetic microbiota community with the data of the stool sample. The metagenomic data were mixed at the reads level while the metaproteomic data were mixed at the MS raw data level. Metagenomics-derived protein sequence databases were constructed using the MD1, MD2, and MD3 pipelines with the annotation tools of BLAST, Kaiju, and Kraken2. The detailed information in terms of the number of genes and species annotated in the protein sequence databases is shown in Supplementary data 6 and Supplementary Table 5. Considering the gene annotation ratio, Kaiju-MD3 remained the best choice. All the annotation strategies based on the MD3 pipeline successfully recovered the 12 species from the mixed metagenomic datasets, while the rankings of the 12 species in the databases considering the number of annotated genes for each species were varied by the different methods (Supplementary Table 6).

**Fig. 5:**
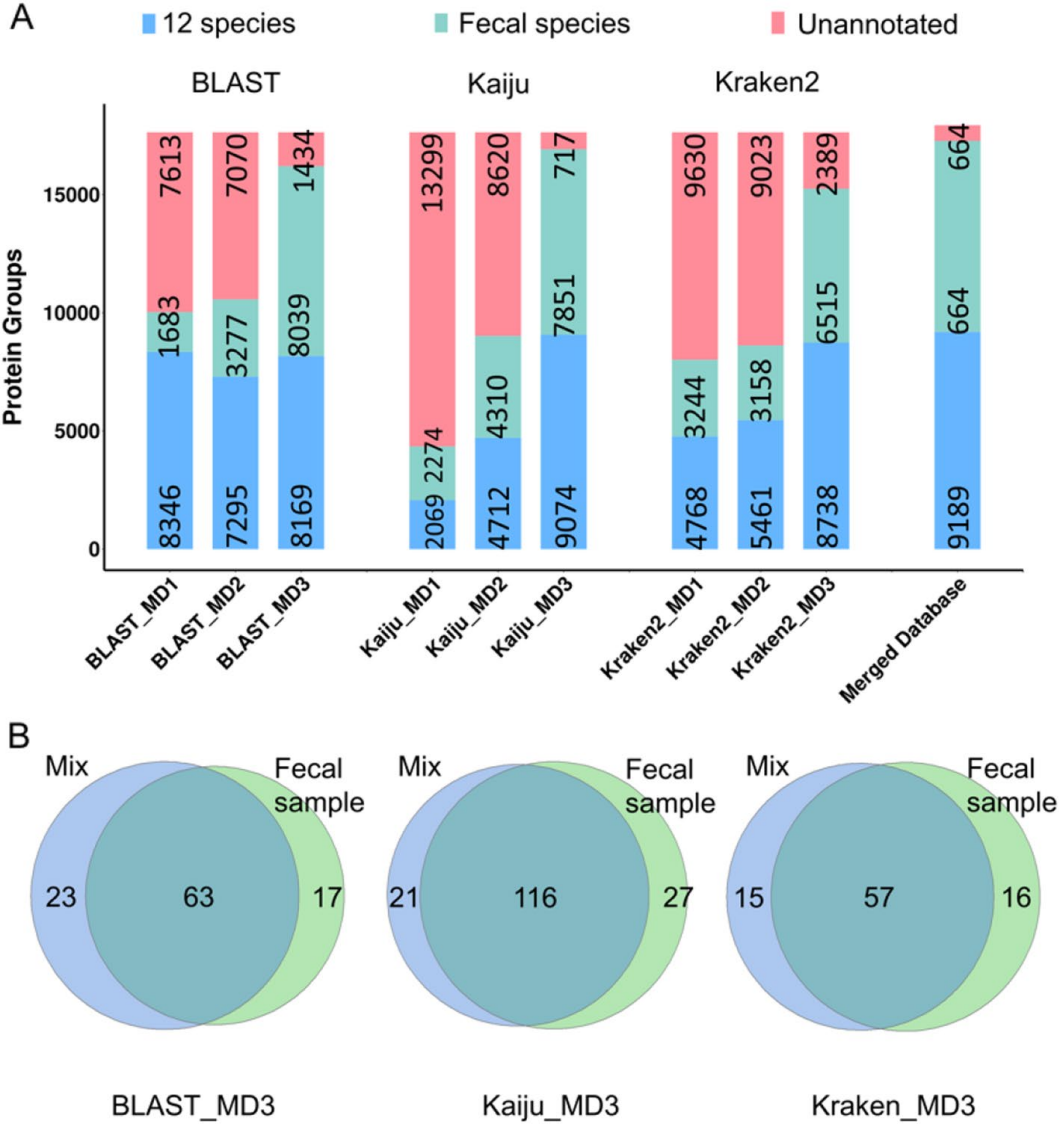
**A** Bar plots of the numbers of protein groups identified from the 12 species of the synthetic microbial community, from the other species, and without taxonomic annotation information. The protein sequence database was generated from the mixed metagenome data of the synthetic microbial community and the fecal sample using the MD1, MD2, and MD3 pipelines with different annotation tools. The MS data were mixed from the synthetic microbial community and the fecal sample. A merged protein sequence database of the 12 species and the fecal sample was used as the reference. **B** Venn diagrams of species identified from the in silico mixed metaproteome dataset and the fecal metaproteome dataset. The protein sequence databases were generated from the mixed metagenome data of the synthetic microbial community and the fecal sample or from the fecal sample only using the MD3 pipeline with different annotation tools

The metagenomics-derived protein sequence databases were then used to analyze the in silico mixed MS data of the synthetic community and the fecal sample. As shown in Fig. 5, Table 2, and Supplementary data 7, in general, the MD3 strategy performed the best and significantly better than the MD1 and MD2. For BLAST, although the numbers of protein groups identified for the 12 species were comparable among the different pipelines of MD1, MD2, and MD3. The numbers of protein groups identified from the other species, i.e., from the fecal sample, were significantly lower with the MD1 and MD2 pipeline compared to the MD3 pipeline. In the mixed data, the 12 species can be viewed as the high abundant ones. Such results indicated that BLAST with MD1 and MD2 pipelines in protein sequence database construction were less sensitive to low abundant species compared to the MD3 pipeline. Among all the methods, the database by MD3 with Kaiju performed the best. We also merged the protein sequence databases of the synthetic microbiota community and the stool sample (merged database) for further comparison, where both databases were built by the MD3 pipeline with the Kaiju annotation using the metagenomic data separately. As shown in Fig. 5 and Table 2, the protein sequence database built from the in silico mixed metagenome data provided a performance similar to the merged database. We have also compared the species identified from the mixed metaproteome data or from the fecal metaproteome data. As shown in Fig. 5B, more than 78% species identified from the fecal sample were recovered from the mixed metaproteome data using the protein sequence database built by the MD3 pipeline with different annotation tools (BLAST, Kraken2, and Kaiju), which further illustrated the robustness and stability of the MD3 pipeline. Among the three tools, Kaiju with MD3 showed the highest recovery (81.1%, 116/143) of species from the fecal sample. All these results demonstrated the effectiveness of the MD3 strategy in protein sequence database construction from metagenome data and again illustrated that the MD3 with Kaiju can provide the optimal taxonomic annotation.

**Table 2:** Identification results of protein groups from the in silico mixed metaproteome data of the synthetic microbial community and the fecal sample using different databases

	BLAST			Kaiju			Kraken2			Merged databases
Species name	MD1	MD2	MD3	MD1	MD2	MD3	MD1	MD2	MD3	
*Bacteroides fragilis*	509	428	**526**	152	421	**523**	511	540	**537**	486
*Citrobacter freundii*	938	756	**670**	123	410	**964**	58	89	**977**	926
*Clostridium butyricum*	696	682	**666**	158	429	**662**	699	699	**669**	640
*Enterobacter asburiae*	906	850	**863**	54	132	**917**	417	740	**922**	889
*Enterococcus casseliflavus*	56	107	**369**	66	356	**384**	80	237	**384**	360
*Enterococcus faecalis*	315	309	**319**	63	197	**315**	303	316	**315**	305
*Escherichia coli*	1075	948	**1059**	148	271	**1164**	174	219	**1080**	1030
*Klebsiella aerogenes*	500	434	**456**	355	460	**506**	489	515	**511**	467
*Klebsiella pneumoniae*	976	683	**863**	218	481	**1277**	109	109	**970**	1811
*Lactobacillus acidophilus*	743	747	**748**	542	748	**748**	742	748	**750**	720
*Morganella morganii*	1155	915	**1153**	171	718	**1131**	1158	1159	**1147**	1096
*Pseudomonas aeruginosa*	477	436	**477**	19	89	**483**	28	90	**476**	459
Target 12 species	8346	7295	**8169**	2069	4712	**9074**	4768	5461	**8738**	9189
Other species	1683	3277	**8039**	2274	4310	**7851**	3244	3158	**6515**	8090
Unannotated	7613	7070	**1434**	13,299	8620	**717**	9630	9023	**2389**	664
Total	17,642	17,642	17,642	17,642	17,642	17,642	17,642	17,642	17,642	17,943

The in silico mixed metagenome data were used to generated the protein sequence databases. A merged protein sequence database of the 12 species and the fecal sample was used as the reference

## Discussion

In this study, we compared three taxonomic annotation pipelines to construct metagenomics-based protein sequence databases and assessed their performance in metaproteomics analysis using a lab-assembled microbial mixture, a real-life stool sample, and the in silico mixed data of the two samples. To date, one widely used approach in constructing metagenomics-based protein sequence databases is based on annotation of predicted individual genes [12, 24, 26] (MD1 in this study). We found that this pipeline could result in a large number of annotated species not actually present in the sample due to inaccurate annotation on short genes. As annotations on long contigs are expected to be more reliable than those on short genes, we further used the second pipeline (MD2) for taxonomic annotation, where the taxonomy information of contigs was passed on to its genes. The risk of this pipeline is that reads from different species can be wrongly assembled to one contig due to the partial sequence similarity among species. As shown in Supplementary Fig. 4, there was indeed a significant portion of contigs with more than one species information. Therefore, we proposed the third annotation pipeline (MD3), named ConDiGA, which first selected the most confident species from the annotation results of contigs, and then performed gene-level annotation against these most confident species. We compared the MD1, MD2, and MD3 annotation strategies with BLAST, Kaiju, and Kraren2 as the annotation tools through the synthetic microbiota community of 12 species. The results generally revealed that the MD3 pipeline outperformed the MD1 and MD2 strategies not only in terms of the numbers of protein groups identified, but also in terms of the sensitivity and accuracy of annotations. As for the three annotation tools, BLAST performed better overall but Kaiju-MD3 managed to become the most efficient strategy. This circumstance remained consistent for the real-life stool sample where the annotation rate of MD3 was significantly higher than that of MD1 and MD2, which proved that the MD3 pipeline remained its advantages when it came to the analysis of complex real-life samples.

To further benchmark the ability to deal with complex samples, we computationally mixed the metagenome and metaproteome data of the synthetic microbiota community and the stool sample and performed annotation by the aforementioned three pipelines. Again, the MD3 pipeline provided the most efficient and robust results. All 12 species were successfully recovered by the MD3 pipeline with different annotation tools. The MD3 combined with Kaiju achieved the largest number of protein groups annotated to the 12 species as well as to the other species from the fecal sample.

## Conclusions

In summary, we proposed an accurate taxonomic annotation pipeline based on metagenomic data for deep metaproteomics analysis, namely contigs directed gene annotation (ConDiGA), and demonstrated the importance of correct gene annotation in constructing protein sequence databases for metaproteomics. In our strategy of ConDiGA, the assembled contigs were firstly annotated and then the most confident species from the annotation results were selected by considering genome coverage as well as taxonomic abundance. The predicted genes were then aligned against the reference genomes of the selected species. As for the performance evaluation of different annotation strategies, we compared current annotation strategies with ConDiGA on a synthetic 12-species microbiota community, a real-life stool sample, and the in silico mixed data of the two samples, using three state-of-the-art annotation tools, i.e., BLAST, Kaiju, and Kraken2. We found that ConDiGA surpassed all other annotation strategies in multiple areas such as annotation coverage, annotation accuracy, and annotation of low-abundance species. Our optimized taxonomic annotation pipeline can tackle the current problem of annotation reliability in metagenomics-derived protein sequence database and can promote the development of metaproteomics. The metagenomic and the metaproteomic data of the 12 species benchmark sample, as well as the ConDiGA pipeline, are publicly available, which can be used for the evaluation of various data analysis pipelines as well as the analysis of microbiota samples.

## Methods

### Bacteria culture and sample collection

The 12 microbial strains (Supplementary Table 1) used in this study were purchased from American Type Culture Collection (ATCC) or China Center of Industrial Culture Collection (CICC). After culture in tryptic soy broth (TSB), brain heart infusion (BHI), or De Man, Rogosa, and Sharpe agar (MRS), the concentrations of bacterial cells were measured using the plate counting method, and the optical density of bacterial solution determined at the wavelength of 595 nm (OD_595_). The bacterial cultures were then washed twice with phosphate-buffered saline (PBS) at pH 7.4 (Solarbio, Beijing, China) and stored at − 80 °C. The 12 bacterial strains were mixed with different cell numbers (Supplementary Table 1) to construct a synthetic microbial community. The fecal sample was collected from a healthy volunteer and stored at − 80 °C for the subsequent experiment.

### Metaproteomic sample preparation

The synthetic microbial community mixture was resuspended in a lysis buffer containing protease inhibitor cocktail (EDTA-free, 1 ×), 20 mM Tris–HCl pH 8.8, 8 M urea, and 1% sodium dodecyl sulfate (SDS) and then sonicated (50 W, 20 Hz, 10 min) using an ultrasonic crusher in an ice bath. Next, the obtained solution was centrifuged (12,000 g, 10 min, 4 °C) to remove cell debris. Proteins in supernatant were quantified using a Pierce BCA assay Kit (Thermo Fisher Scientific, Waltham, MA, USA) prior to lyophilization. For proteolysis, 300 μg of proteins were dissolved in 300 μL 8 M urea and 6.1 μL 0.5 M Tris-(2-carboxyethyl) phosphine (TCEP), followed by incubation for 1 h at 37 °C with shaking at 600 rpm. Following this, 18 μL iodoacetamide (IAA, 0.5 M) solution was added into the dissolved protein solution and incubated at room temperature for 45 min (in darkness). The proteins were precipitated using 1.5-mL pre-cooled acetone (− 20 °C) for 4 h and washed twice with the pre-cooled acetone. After drying at room temperature, the proteins were redissolved in triethylammonium bicarbonate (TEAB) solution (200 μL, 0.1 M). For trypsin digestion, 6 μg of trypsin (Beijing Hualishi Technology Ltd, Beijing, China) was added into the protein solution and incubated for 16 h at 37 °C with shaking at 600 rpm. Finally, the obtained peptides were transferred to MonoSpin C18 column (Tokyo Japan GL Sciences Inc) for desalting. After the desalting, peptides were quantified by Pierce quantitative colorimetric peptide assay (Thermo Fisher Scientific, Waltham, MA, USA).

For human stool sample processing, differential centrifugation was used to enrich microbial cells according to a previous report [7, 32]. Specifically, 0.5-g stool sample was mixed with 20 mL PBS and shaken for 30 min at 25 °C and 100 rpm. Then, the suspension was first centrifuged at 500 g, 4 °C for 5 min to remove the large particles. Next, the supernatant was collected and subjected to high-speed centrifugation (12,000 g, 10 min) to collect the precipitates. The precipitates from the 500 g centrifugation were subjected to the differential centrifugation strategy again. Then, the two precipitates from the 12,000 g centrifugation were combined as the final collected microbes. For protein extraction, the collected microbial precipitates were processed by liquid nitrogen grinding. The milled powder was dissolved in 0.5 mL lysis solution (100 mM dithiothreitol (DTT), 2% SDS, and 20 mM Tris–HCl pH 8.8). After heating at 95 °C for 30 min, the dissolved protein solution was centrifugated at 12,000 g, 4 °C for 10 min to collect the supernatants. To remove SDS, pre-cooled acetone solution (− 20 °C) was added and incubated under − 20 °C for 4 h. The precipitated proteins were collected by centrifugation and then washed twice using pre-cooled acetone. The obtained proteins were dried at room temperature and dissolved in 0.5-mL lysis buffer. The protein quantification method and proteolysis procedure were the same as those for the synthetic microbial community aforementioned.

### LC–MS/MS analysis

For each sample, 10 μg of peptides was redissolved in 30 μL solvent A (0.1% formic acid in water) and analyzed by a nanoESI timsTOF pro mass spectrometer (Bruker, Bremen, Germany) with a nanoElute® (Bruker Daltonics) LC system. For each injection of the timsTOF pro MS analysis, 200-ng peptides were separated by a C18 reversed phase analytical column with 1.6 μm resin (25 cm × 75 μm i.d., Ionopticks) by a 120-min gradient with phase A as 0.1% formic acid in water and phase B as 0.1% formic acid in 99.9% acetonitrile (Supplementary Table 7). The column flow rate was maintained at 300 nL/min with a column temperature of 50 °C. The instrument was operated in the data-dependent acquisition-parallel accumulation serial fragmentation (DDA-PASEF) mode with 10 PASEF scans per topN acquisition cycle and accumulation ramp times of 100 ms each. MS and MS/MS spectra were recorded from 100 to 1700 m/z with an ion mobility range (1/K0) of 0.7–1.3 versus/cm^2^. Charge was set to 0–5. The “target value” was set to 10,000. The dynamic exclusion was activated and set to 0.4 min. The quadrupole isolation width was set to 2 Th for m/z < 700 and 3 Th for m/z > 700.

### Metagenomic sequencing and data analysis

DNA was extracted from the synthetic microbial community or the stool sample using HiPure Bacterial DNA Kits (Magen, Guangzhou, China) according to the manufacturer’s instructions. The quality of the extracted DNA was detected using Qubit (Thermo Fisher Scientific, Waltham, MA) and Nanodrop (Thermo Fisher Scientific, Waltham, MA). The extracted genomic DNA was firstly fragmented by sonication to a size of around 350 bp, then end-repaired, A-tailed, and adaptor ligated using NEBNext ΜLtra DNA Library Prep Kit for Illumina (NEB, Ipswich, MA, USA) according to the manufacturer’s preparation protocol. DNA fragments with a length of 300–400 bp were enriched by PCR. The PCR products were purified using an AMPure XP system (Beckman Coulter, Brea, CA, USA). The libraries were analyzed for size distribution by a 2100 Bioanalyzer (Agilent, Santa Clara, CA, USA) and quantified using real-time PCR. Genome sequencing was performed on an Illumina Novaseq 6000 sequencer (Illumina, Inc., San Diego, CA, USA) using the pair-end technology.

Raw reads from the Illumina platform were filtered using FASTP (version 0.18.0) with the following criteria: (1) removing reads with ≥ 10% unidentified nucleotides; (2) removing reads with ≥ 50% bases having phred quality scores ≤ 20; (3) removing reads aligned to the barcode adapter. After filtering, resulted clean reads were assembled using MEGAHIT (version 1.2.9) [33] (with the parameters k-min = 21 and k-max = 141). Genes were predicted based on the final assembled contigs using MetaGeneMark (version 3.38) [34] with the default parameters. The same method was applied to the synthetic microbial community dataset and the stool sample dataset.

### Taxonomic annotation and protein sequence database construction for metaproteomic analysis

In MD1, the predicted genes were directly annotated using the taxonomic classification tools of BLAST, Kraken2, or Kaiju. Nucleotide BLAST (blastn version 2.13.0 +) was run with the parameters: -outfmt "6 qseqid sseqid staxids sscinames scomnames sskingdoms pident length qlen slen mismatch gapopen gaps qstart qend sstart send stitle evalue bitscore qcovs qcovhsp" and -max_target_seqs 1. The NCBI BLAST nt database was downloaded on 17 January 2023 and was used for the BLAST annotation. The command used included the following: blastn -db < db_path > -query final.contigs.fa -out final.contigs.fa.blastn.results.out -num_threads 32 -outfmt "6 qseqid sseqid staxids sscinames scomnames sskingdoms pident length qlen slen mismatch gapopen gaps qstart qend sstart send stitle evalue bitscore qcovs qcovhsp" -max_target_seqs 1. The standard Kraken2 index databases from https://benlangmead.github.io/aws-indexes/k2 were used. Kraken2 (version 2.1.1) command used included the following: kraken2 –threads 56 –db < db_path > –use-names –output kraken_res_0.1.txt –confidence 0.1 –report kraken_report_0.1.txt final.contigs.fa. As the Kraken2 uses a k-mer-based approach to individually annotate the predicted genes, it can introduce mistakes and result in a large number of annotated species not existing in the sample. To avoid this issue, the confidence parameter of Kraken2 was increased from 0 (default value) to 0.1. The Kaiju Web server (available from https://kaiju.binf.ku.dk/server) was used with default parameters and the NCBI BLAST nr reference database was selected.

In MD2, the assembled contigs were annotated instead of predicted genes using BLAST, Kraken2, or Kaiju with the same parameters as for MD1. Then, genes were annotated according to the species label of the contigs they belong to.

In MD3, the assembled contigs were annotated using BLAST, Kraken2, or Kaiju with the same parameters as for MD1. For the synthetic microbial community, the species with relative sequence abundance > 0.5% and coverage ≥ 0.1% were selected as the most confident species. For the fecal sample and for the in silico mixed data, the species with a relative sequence abundance ≥ 0.01% and genome coverage ≥ 0.1% were selected as the most confident species. Then, the predicted genes were annotated to one of the selected most confident species based on the best alignment from Minimap2 [35].

To build the Meta-PA database, we first predict the genes from the contigs using MetaGeneMark. The amino acid sequences of the predicted genes were annotated using the UniProtKB/TrEMBL database (downloaded on 15th of December 2022 from https://www.uniprot.org/help/downloads) using Protein BLAST (BLASTP version 2.13.0 +).

To build the Meta-6FT dataset, we first processed the assembled contigs in an alternative way based on naïve six-frame translation. The Sequence Processor and Translator script from https://cgpdb.ucdavis.edu/DNA_SixFrames_Translation/ was used to obtain the naïve six-frame translation. The command used included the following: python seqs_processor_and_translator_bin_V118_AGCT.py final.contigs.fa final.contigs.xout DNA 6 1 BIN 24. Results from all the six frames were combined to form one file meta.6FT.faa. Then, we used MMseqs2 [30] (version 13.45111) to align sequences to the UniProtKB/TrEMBL database. The command used included the following: mmseqs createdb meta.6FT.faa target_seq; mmseqs search target_seq TrEMBL_mmseqs/uniprot_trembl_db results./tmp/; mmseqs createtsv target_seq TrEMBL_mmseqs/uniprot_trembl_db results meta.6FT_annot.tsv –full-header. Each gene was annotated with the species having the longest alignment with over 10% alignment fraction and over 10% sequence identity.

### Metaproteome data analysis and bioinformatic analysis

Uniprot-based databases were built from the reference proteome of the used microbes at the species level, genus level, or family level. For species level Uniprot-based database, the reference stains for each species shown in Supplementary Table 3 were used to construct the database, while for the genus-level or family-level Uniprot-based databases, all strains included in the corresponding genus or family were used to construct the databases. The metagenomics-based databases were translated from the annotated genes using MetaGeneMark (version 3.38) [34]. Database searching for all metaproteomic data was carried out using PEAKS Studio (version X pro, Bioinformatics Solutions Inc., Canada). The metaproteomic data were searched with the following parameters: precursor ion tolerance 15 ppm, fragment ion tolerance 0.05 Da, maximum of 2 missed cleavage sites, carbamidomethylation (C) of cysteine as fixed modification, oxidation of methionine and deamidation (NQ) as variable modification, trypsin as proteolytic enzyme, and 1% false discovery rate threshold at both peptide and protein group level. KEGG annotation was performed using the Ghost-KOALA web server. Data visualization was conducted with R (version 3.5.1, https://www.r-project.org/) using the packages of ggplot2 (https://github.com/tidyverse/ggplot2) and AntV G2 (https://github.com/antvis/g2).

### Supplementary Information

**Additional file 1: Supplementary Table 1.** The composition of the simulated communities with 12 species. **Supplementary Table 2.** Characteristics of the protein sequence databases derived from the metagenomic data of the synthetic microbial community. **Supplementary Table 3.** Details of the reference proteome of the 12 species. **Supplementary Table 4.** Characteristics of protein sequence databases derived from the metagenomic data of the stool sample. **Supplementary Table 5.** Characteristics of protein sequence databases derived from the in-silico mixed metagenomic data of the synthetic microbial community and the fecal sample. **Supplementary Table 6.** Abundance ranking of the 12 species in MD3-based protein sequence databases using the annotation tools of BLAST, Kaiju and Kraken2 from the in-silico mixed metagenomic data. **Supplementary Table 7.** LC gradient.

**Additional file 2. Supplementary Data 1. **Annotation of the genes from the synthetic microbial community of 12 species using different taxonomic classification tools and strategies.

**Additional file 3. Supplementary Data 2. **Identification result of protein groups and peptides from the synthetic microbial community using different databases.

**Additional file 4. Supplementary Data 3. **Annotation of genes from the stool sample using different taxonomic classification tools and strategies.

**Additional file 5. Supplementary Data 4. **Identification result of protein groups and peptides from the stool sample using different databases.

**Additional file 6. Supplementary Data 5. **The relative taxonomic abundance at the metaproteomic (MP) level and at the metagenomic (MG) level.

**Additional file 7. Supplementary Data 6.** Annotation of the genes from the in-silico mixed data using different taxonomic classification tools and strategies.

**Additional file 8. Supplementary Data 7.** Identification result of protein groups and peptides from the in-silico mixed metaproteomic data using different databases.

## Data Availability

All raw MS data, spectral libraries, and search results generated in this study have been deposited to the ProteomeXchange via the iProX [36] partner repository with accession numbers PXD034815 or IPX0004588000. The DNA-seq data have been deposited in the Sequence Read Archive (SRA) database, with accession PRJNA879328 (https://www.ncbi.nlm.nih.gov/bioproject/PRJNA879328). The steps and scripts used to construct the different metagenomic-based databases are available on GitHub at https://github.com/metagentools/metagenomic-database-utilities. The code of ConDiGA pipeline can be found at https://github.com/metagentools/ConDiGA. The “assembly_summary.txt” file used for the experiments in the manuscript was downloaded from NCBI (https://ftp.ncbi.nlm.nih.gov/genomes/genbank/bacteria/assembly_summary.txt) on the 26th of May 2021.
