# Supplementary material for: Contigs directed gene annotation (ConDiGA) for accurate protein sequence database construction in metaproteomics
Source: Microbiome. 2024 Mar 19;12:58. doi: 10.1186/s40168-024-01775-3 (PMC10949615; doi:10.1186/s40168-024-01775-3)
Supplement: Supplementary file 2 — Additional file 1: Supplementary Table 1. The composition of the simulated communities with 12 species. Supplementary Table 2. Characteristics of the protein sequence databases derived from the metagenomic data of the synthetic microbial community. Supplementary Table 3. Details of the reference proteome of the 12 species. Supplementary Table 4. Characteristics of protein sequence databases derived from the metagenomic data of the stool sample. Supplementary Table 5. Characteristics of protein sequence databases derived from the in-silico mixed metagenomic data of the synthetic microbial community and the fecal sample. Supplementary Table 6. Abundance ranking of the 12 species in MD3-based protein sequence databases using the annotation tools of BLAST, Kaiju and Kraken2 from the in-silico mixed metagenomic data. Supplementary Table 7. LC gradient. [file 40168_2024_1775_MOESM1_ESM.docx]

Supplementary information for

**Contigs directed gene annotation (ConDiGA) for accurate protein sequence database construction in metaproteomics**

Wu et al.

**Supplementary Table 1.** The composition of the simulated communities with 12 species.

| **Strain** | **Cell number (10^8^ CFU)** |
| --- | --- |
| *Morganella morganii* CICC 21517 | 20 |
| *Pseudomonas aeruginosa* ATCC 27853 | 4 |
| *Klebsiella pneumonia* ATCC 4352 | 20 |
| *Citrobacter freundii* CICC 10404 | 4 |
| *Enterococcus faecalis* ATCC 19433 | 2 |
| *Klebsiella aerogenes* ATCC 13048 | 2 |
| *Bacteroides fragilis* ATCC 25285 | 2 |
| *Enterobacter asburiae* ATCC 35953 | 10 |
| *Enterococcus casseliflavus* ATCC 700327 | 4 |
| *Escherichia coli* ATCC 25922 | 1.32 |
| *Clostridium butyricum* ATCC 1939 | 0.067 |
| *Lactobacillus acidophilus* CICC 6074 | 0.333 |

**Supplementary Table 2.** Characteristics of the protein sequence databases derived from the metagenomic data of the synthetic microbial community.

| **Database** | **Total no. of genes available** | **No. of genes annotated to 12 species** | **No. of genes annotated to other species** | **No. of genes unannotated** | **No. of species found** |
| --- | --- | --- | --- | --- | --- |
| MD1_Kraken2 | 63 010 | 30 053 (47.70%) | 4344 (6.89%) | 28 613 | 52 |
| MD2_Kraken2 | 63 010 | 34 133 (54.17%) | 3974 (6.26%) | 24 930 | 33 |
| MD3_Kraken2 | 63 010 | 52 055 (82.61%) | 0 | 10 955 | 12 |
| MD1_Kaiju | 63 010 | 18 534 (29.41%) | 1868 (2.96%) | 42 608 | 157 |
| MD2_Kaiju | 63 010 | 31 269 (49.63%) | 2266 (3.60%) | 29 475 | 121 |
| MD3_Kaiju | 63 010 | 54 646 (86.73%) | 0 | 8364 | 12 |
| MD1_BLAST | 63 010 | 48 331 (76.70%) | 8846 (14.04%) | 5833 | 122 |
| MD2_BLAST | 63 010 | 44 937 (71.32%) | 6828 (10.84%) | 11 245 | 77 |
| MD3_BLAST | 63 010 | 46 610 (74.00%) | 5869 (9.31%) | 10 531 | 12 |
| Meta-PA | 63 010 | 16 785 (26.64%) | 9250 (14.68%) | 36 975 | 1160 |
| Meta-6FT | 49 104^*^ | 7312 (14.89%) | 13 195 (26.87%) | 28 597 | 1887 |

^*^ The naive six-frame translation yielded a smaller number of genes than the MetaGeneMark, because MetaGeneMark has been optimized better to predict genes in metagenomes than the naive six-frame translation which can be affected during ORF identification.

**Supplementary Table 3.** Details of the reference proteome of the 12 species.

| **Strain** | **Protein count** | **Uniprot Proteome ID/ Organism ID** |
| --- | --- | --- |
| *Escherichia coli* ATCC 25922 | 5062 | UP000000558/83334 |
| *Citrobacter freundii* CICC 10404 | 5149 | UP000305402/ 546 |
| *Enterococcus casseliflavus* ATCC 700327 | 3112 | UP000012675/ 565655 |
| *Enterococcus faecalis* ATCC 19433 | 3240 | UP000001415/ 226185 |
| *Pseudomonas aeruginosa* ATCC 27853 | 5564 | UP000002438/ 208964 |
| *Enterobacter asburiae* ATCC 35953 | 5254 | UP000216915/ 61645 |
| *Klebsiella aerogenes* ATCC 13048 | 4909 | UP000008881/ 1028307 |
| *Klebsiella pneumonia* ATCC 4352 | 5126 | UP000000265/ 272620 |
| *Morganella morganii* CICC 21517 | 3510 | UP000011834/ 1124991 |
| *Bacteroides fragilis* ATCC 25285 | 4234 | UP000006731/ 272559 |
| *Lactobacillus acidophilus* CICC 6074 | 1859 | UP000006381/ 272621 |
| *Clostridium butyricum* ATCC 1939 | 4245 | UP000003081/ 632245 |

**Supplementary Table 4.** Characteristics of protein sequence databases derived from the metagenomic data of the stool sample.

| **Database** | **Total no. of genes available** | **No. of genes annotated** | **No. of genes unannotated** | **No. of species found** |
| --- | --- | --- | --- | --- |
| MD1_Kraken2 | 468 096 | 142 549 (30.45%) | 325 547 | 428 |
| MD2_Kraken2 | 468 096 | 151 017 (32.26%) | 317 079 | 229 |
| MD3_Kraken2 | 468 096 | 168 571 (36.01%) | 299 525 | 73 |
| MD1_Kaiju | 468 096 | 179 070 (38.26%) | 289 026 | 3168 |
| MD2_Kaiju | 468 096 | 240 658 (51.41%) | 227 438 | 2985 |
| MD3_Kaiju | 468 096 | 284 894 (60.86%) | 183 202 | 155 |
| MD1_BLAST | 468 096 | 114 885 (24.54%) | 353 211 | 3298 |
| MD2_BLAST | 468 096 | 119 011 (25.42%) | 349 085 | 2372 |
| MD3_BLAST | 468 096 | 209 117 (44.67%) | 258 979 | 90 |

**Supplementary Table 5.** Characteristics of protein sequence databases derived from the *in-silico* mixed metagenomic data of the synthetic microbial community and the fecal sample.

| **Database** | **Total no. of genes available** | **No. of genes annotated to 12 species** | **No. of genes annotated to other species** | **No. of genes unannotated** | **No. of species found** |
| --- | --- | --- | --- | --- | --- |
| MD1_Kraken2 | 526 234 | 32 313 (6.14%) | 141 707 (26.93%) | 352 214 | 495 |
| MD2_Kraken2 | 526 234 | 35 888 (6.82%) | 14 9815 (28.47%) | 340 531 | 259 |
| MD3_Kraken2 | 526 234 | 52 961 (10.06%) | 169 852 (32.28%) | 345 748 | 72 |
| MD1_Kaiju | 526 234 | 17 162 (3.26%) | 147 552 (28.04%) | 361 520 | 3322 |
| MD2_Kaiju | 526 234 | 32 367 (6.15%) | 239 894 (45.59%) | 253973 | 3111 |
| MD3_Kaiju | 526 234 | 59 547 (11.32%) | 273 333 (51.94%) | 193 354 | 144 |
| MD1_BLAST | 526 234 | 52 840 (10.04%) | 112 449 (21.37%) | 360 945 | 3443 |
| MD2_BLAST | 526 234 | 46 591 (8.85%) | 118 498 (22.52%) | 361 145 | 2377 |
| MD3_BLAST | 526 234 | 49 980 (9.50%) | 215 211 (40.90%) | 261 043 | 101 |

**Supplementary Table 6.** Abundance ranking of the 12 species in MD3-based protein sequence databases using the annotation tools of BLAST, Kaiju and Kraken2 from the *in-silico* mixed metagenomic data.

| **Species** | **Kraken** | **Kaiju** | **BLAST** |
| --- | --- | --- | --- |
| *Bacteroides fragilis* | 5 | 6 | 8 |
| *Enterococcus casseliflavus* | 17 | 14 | 22 |
| *Klebsiella pneumoniae* | 14 | 9 | 12 |
| *Citrobacter freundii* | 16 | 8 | 9 |
| *Enterococcus faecalis* | 7 | 10 | 11 |
| *Lactobacillus acidophilus* | 23 | 32 | 29 |
| *Clostridium butyricum* | 3 | 3 | 2 |
| *Escherichia coli* | 13 | 17 | 10 |
| *Morganella morganii* | 2 | 2 | 3 |
| *Enterobacter asburiae* | 8 | 23 | 13 |
| *Klebsiella aerogenes* | 4 | 4 | 7 |
| *Pseudomonas aeruginosa* | 6 | 11 | 1 |

**Supplementary Table 7.** LC gradient.

| **Time (min)** | **B (%)** |
| --- | --- |
| 0 | 2 |
| 105 | 22 |
| 110 | 35 |
| 115 | 80 |
| 120 | 80 |


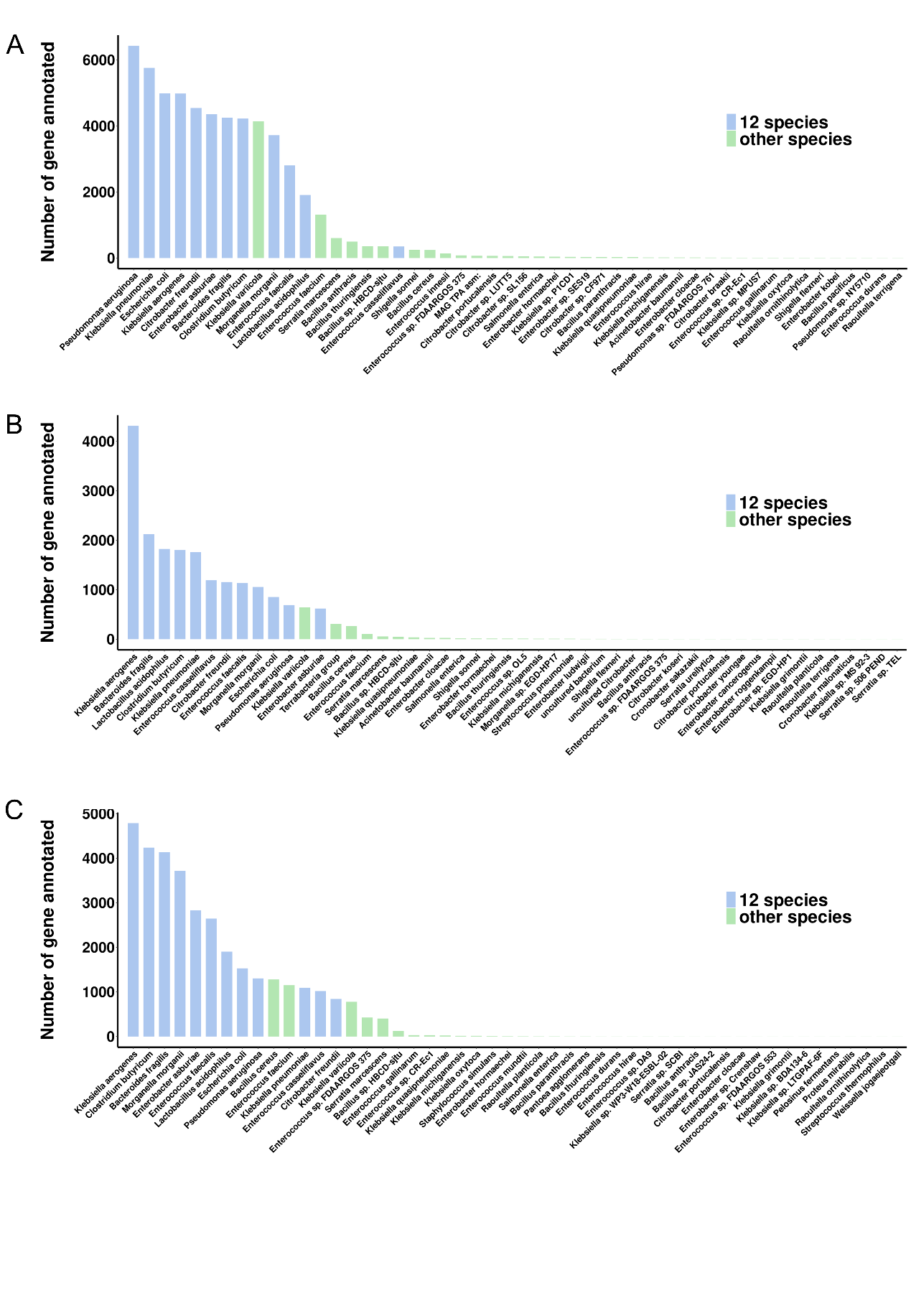


**Supplementary Figure 1.** Numbers of genes annotated for the top 50 species by **(A)** BLAST_MD1, **(B)** Kaiju_MD1 and **(C)** Kraken2_MD1 from the metagenomic sequencing result of the simulated microbial community of the 12 species.


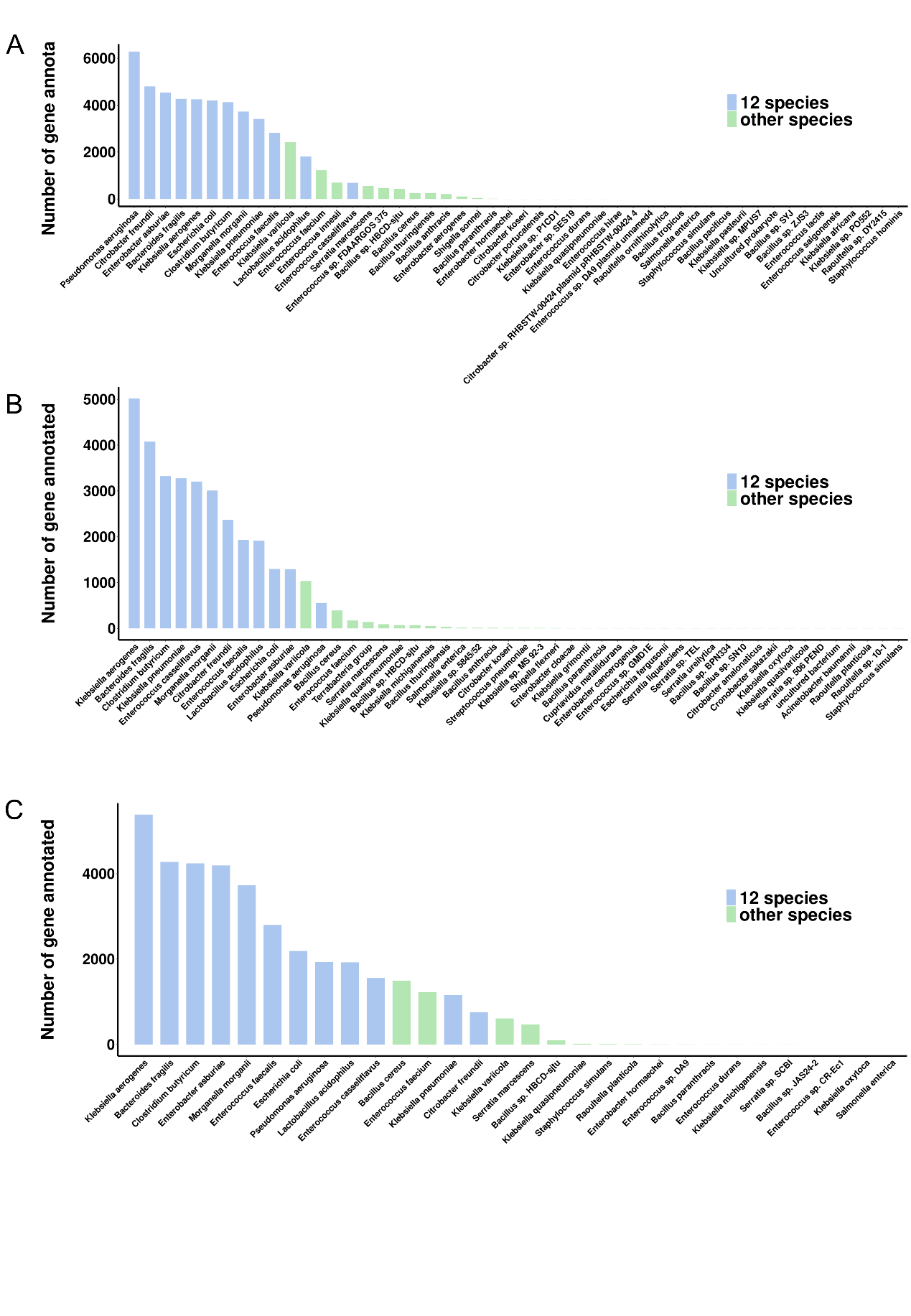


**Supplementary Figure 2.** Numbers of genes annotated for the top 50 species by **(A)** BLAST_MD2, **(B)** Kaiju_MD2 and **(C)** Kraken2_MD2 from the metagenomic sequencing result of the simulated microbial community of the 12 species.

**
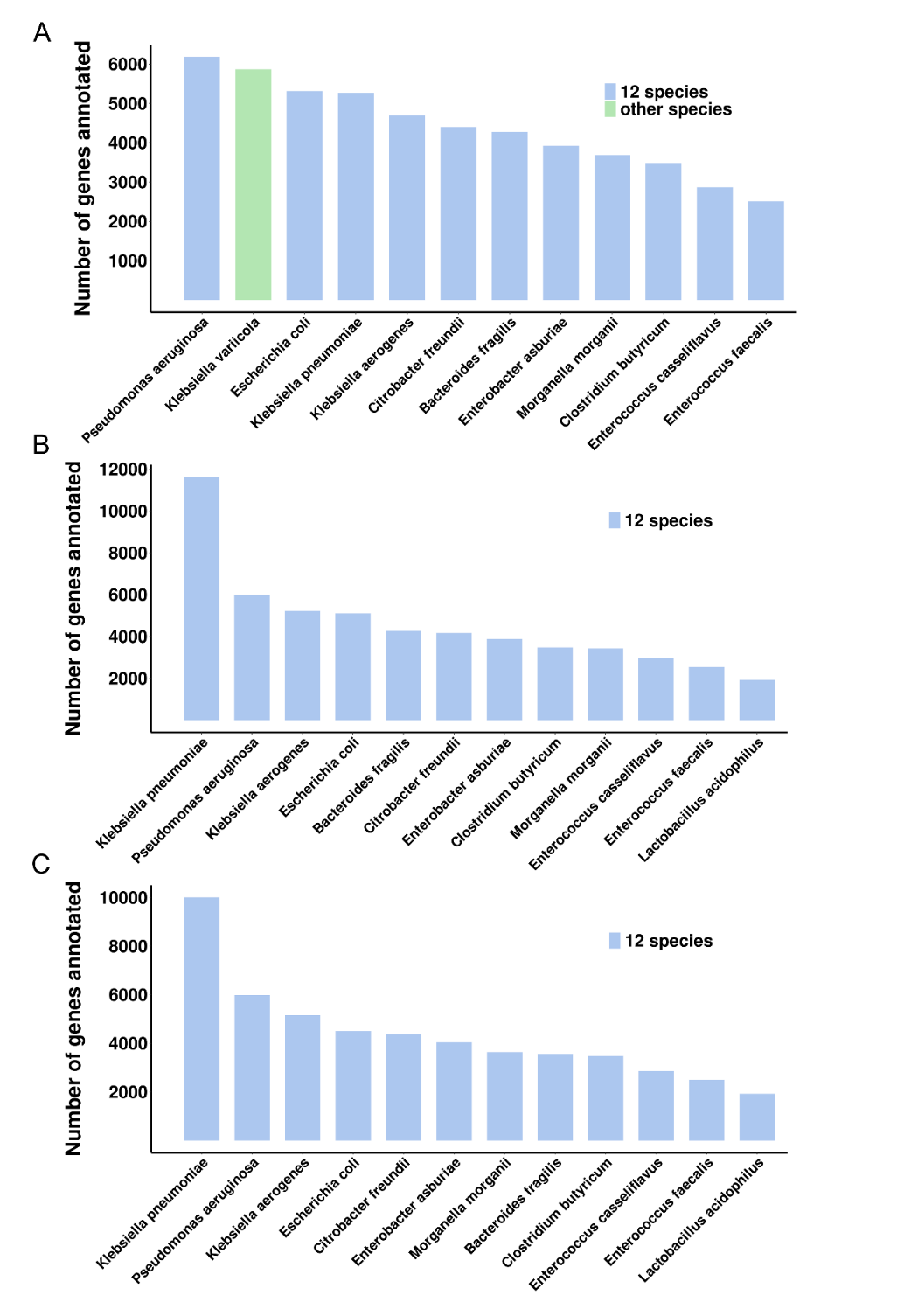
**

**Supplementary Figure 3.** Numbers of genes annotated by **(A)** BLAST_MD3, **(B)** Kaiju_MD3 and **(C)** Kraken2_MD3 from the metagenomic sequencing result of the simulated microbial community of the 12 species.


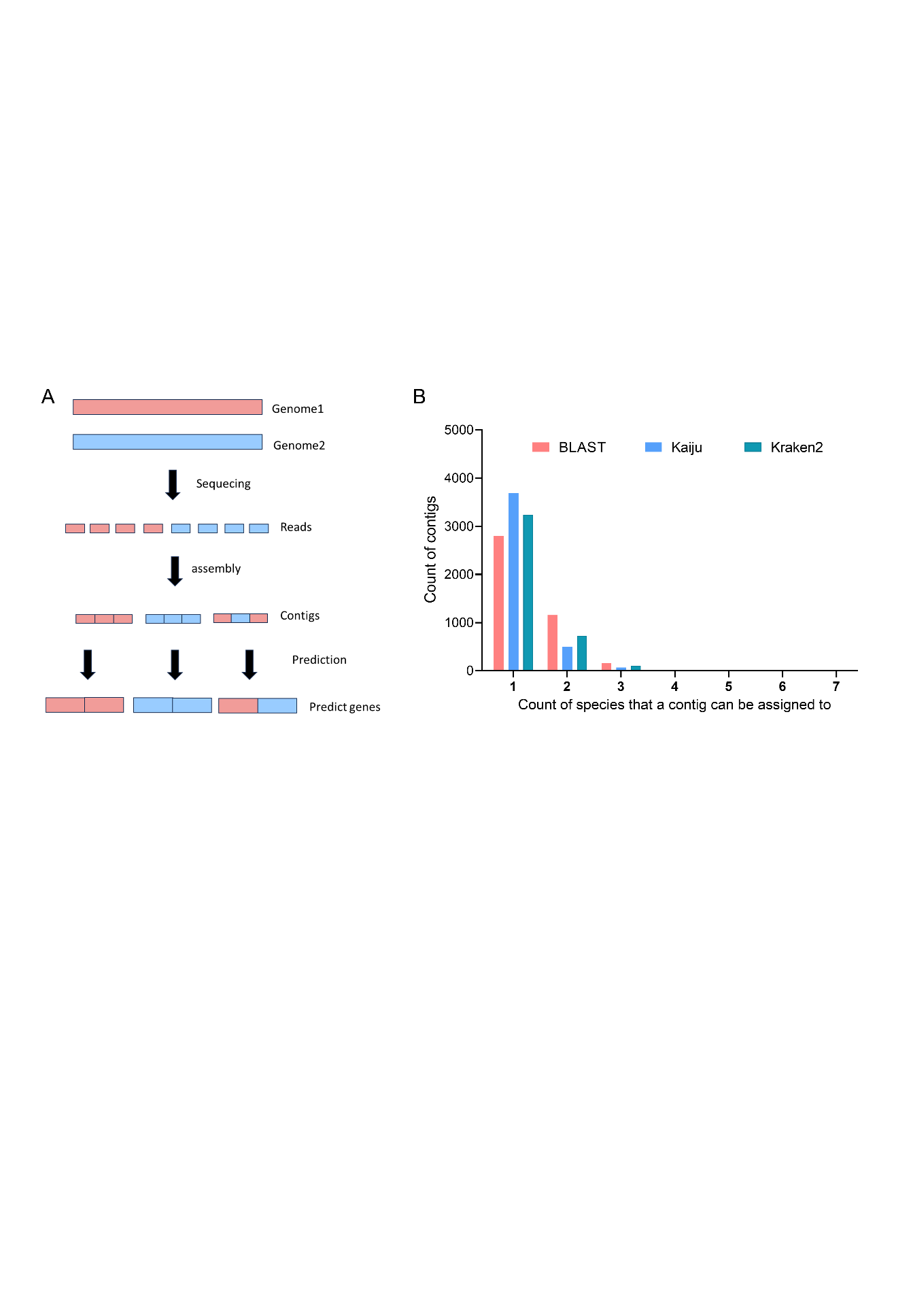


**Supplementary Figure 4.** (A) Schematic illustration of the errors in reads assembly that lead to mistakes in taxonomic annotation. (B) Count of species in one contig by the MD3 pipeline with the different annotation tools based on the metagenomic sequencing results of the simulated microbial community of 12 species.
